# The effectiveness of combined training model in perioperative nursing for implant-based breast reconstruction: a quasi-experimental study

**DOI:** 10.3389/fonc.2026.1794390

**Published:** 2026-06-15

**Authors:** Xiaofeng Mei, Mengqin Zhao, Yi Li, Lili Ge

**Affiliations:** 1 Cancer Center, Department of Breast Surgery, Zhejiang Provincial People’s Hospital (Affiliated People’s Hospital), Hangzhou Medical College, Hangzhou, China; 2 Nursing Department, Zhejiang Provincial People’s Hospital (Affiliated People’s Hospital), Hangzhou Medical College, Hangzhou, China

**Keywords:** breast neoplasms, combined training model, implants, perioperative nursing, quasi-experimental study

## Abstract

**Background:**

Implant-based breast reconstruction demonstrates notable psychological benefits, particularly in enhancing postoperative emotional well-being and self-perception among breast cancer survivors. However, its perioperative nursing process remains inconsistent, often relying on subjective clinical experience. There is a need for a standardized, evidence-based nursing protocol supported by effective training strategies to improve patient outcomes.

**Objective:**

This study aimed to develop and implement a perioperative nursing protocol for implant-based breast reconstruction, supported by a combined training program, and to evaluate its impact on patient satisfaction and postoperative complications.

**Methods:**

A quasi-experimental study was conducted. A nursing protocol was developed using literature review, Delphi consultation, and Analytic Hierarchy Process (AHP), covering preoperative assessment, intraoperative cooperation, and postoperative care. To standardize perioperative nursing procedures, we implemented a structured training program comprising model-based teaching, scenario-based simulations, and hands-on clinical practice. A total of 82 patients were enrolled, with 40 assigned to the experimental group and 42 to the control group. Outcome measures included satisfaction with breast appearance, satisfaction with surgical outcomes, satisfaction with nursing care, the incidence of postoperative complications and the grade of complication. Statistical analyses were conducted using the chi-square test, Fisher’s exact test, Mann-Whitney U test and the independent t-test.

**Results:**

The experimental group demonstrated significantly higher levels of satisfaction across all measured domains compared to the control group (breast satisfaction: 85.37 ± 5.45 vs. 80.32 ± 4.97; surgical outcome satisfaction: 83.66 ± 5.70 vs. 79.23 ± 5.21; care satisfaction: 88.72 ± 6.28 vs. 83.56 ± 5.99; *P* < 0.001). The 30-day postoperative complication incidence was 14.3% in the control group and 5.0% in the experimental group. Although a higher complication proportion was observed in the control group, no statistically significant difference was detected between the two groups (*P*>0.05).The distribution of Clavien-Dindo complication grades showed no statistically significant difference between the two groups (*P*>0.05).

**Conclusions:**

Preliminary findings show the combined training model elevated patient satisfaction and tended to lower postoperative complications. This exploratory method is clinically viable and lays groundwork for subsequent large-scale and multicenter researches.

## 1 Introduction

Breast cancer (BC) is the most prevalent malignancy among women globally, with the highest incidence rates reported in the majority of countries (1). Surgical treatment remains a cornerstone of comprehensive breast cancer management. For patients who are not candidates for breast-conserving surgery, total mastectomy results in the loss of the breast and associated chest wall defects, which can significantly compromise secondary sexual characteristics, diminish self-image, and reduce self-esteem. These changes have a profound impact on patients’ physical and psychological well-being, ultimately reducing their overall quality of life (2, 3). As a result, surgical strategies for breast cancer have gradually evolved from radical resection alone toward approaches that also prioritize postoperative body contour restoration and improvements in psychosocial outcomes. Breast reconstruction, without affecting cancer prognosis or recurrence monitoring, offers the opportunity to restore breast symmetry and improve both physical recovery and psychological resilience in affected patients (4).

Reconstruction techniques are broadly categorized into autologous tissue reconstruction and implant-based breast reconstruction (IBBR). Compared to autologous methods, IBBR is associated with shorter operative time, reduced hospitalization, and greater predictability in aesthetic outcomes. It is particularly suitable for patients with limited physiological tolerance or those unwilling to undergo more complex procedures (5). Despite these advantages, patients undergoing IBBR often experience a range of perioperative stressors, including preoperative anxiety, postoperative pain, restricted mobility, and concerns about reconstructive results. These challenges can hinder recovery and affect the overall patient experience. However, clinical nursing practices in the perioperative care of IBBR patients remain insufficiently standardized and lack comprehensive pathway guidance (6). Specifically, current limitations include inadequate preoperative assessment, non-targeted nursing interventions, and underdeveloped rehabilitation support systems—all of which may compromise the quality of care and patient satisfaction.

In recent years, the importance of integrating structured training into clinical nursing practice has gained attention (7). Multimodal training strategies—such as model-based teaching, scenario-based simulation, and hands-on practice—have demonstrated effectiveness in enhancing clinical performance and patient-centered care (8). However, evidence is limited regarding the impact of such training in the specific context of IBBR nursing.

This study aimed to develop a standardized perioperative nursing protocol for IBBR, supported by an integrated training plan for surgical nurses. Furthermore, the study sought to evaluate the effectiveness of this combined intervention in improving postoperative patient satisfaction and reducing complication rates. By addressing known gaps in nursing consistency and quality, this research aims to contribute to the optimization of care pathways in breast reconstruction surgery.

## 2 Methods

### 2.1 Design

This study consisted of two phases: the development of the integrated training model and the intervention phase. During the development phase, a perioperative nursing protocol and training plan for IBBR were established based on evidence from the literature and a Delphi expert consultation. In the intervention phase, a randomized controlled trial design was not feasible in this clinical setting. This study was conducted at a single center with only one breast surgery ward. To avoid contamination bias between groups, patients were enrolled and grouped strictly according to their hospital admission time. A two-arm quasi-experimental design was employed to assess the effectiveness of the model among eligible participants, who were allocated to either the intervention group or the control group. We monitored potential temporal confounders throughout the entire study period to control time-related bias, including: (1) stable personnel structure with no major staff turnover; and (2) hospital admission, treatment and discharge management policies. All above confounding factors remained stable across the two study stages, effectively minimizing the risk of temporal bias and ensuring the comparability of the two groups.

### 2.2 Participants

The inclusion criteria were as follows:(1) Diagnosis confirmed according to the Guidelines for breast cancer diagnosis and treatment by China Anti-cancer Association (2024 edition) issued by the Chinese Anti-Cancer Association (9), supported by imaging examinations such as ultrasound, mammography, or MRI, and verified by pathological findings;(2) Female patients aged 18 years or older;(3) Candidates for IBBR (either immediate or delayed);(4) Complete clinical records available;(5) Clear consciousness, with the ability to read and communicate effectively. The exclusion criteria were as follows:(1) Voluntary discharge before completion of treatment;(2) Voluntary withdrawal from the study midway;(3) Loss to follow-up.

The study adhered to the ethical principles outlined in the Declaration of Helsinki. At each stage, participants were fully informed of the study’s objectives and procedures. Written and verbal informed consent was obtained from all individuals prior to participation. Ethical approval for the study was granted by the Ethics Committee of Zhejiang Provincial People’s Hospital (Approval No.[ZJPPHEC2023I(186)]).

### 2.3 Grouping method

This study employed a quasi-experimental design. The sample size was calculated using the formula for comparing the means of two independent samples:


$$\text{n}1=n2=2{\left[\frac{(\text{t}\alpha /2+t\beta )S}{\text{δ}}\right]}^{2}$$

With a significance level of α = 0.05 (two-sided) and statistical power of 1 − β = 0.90, the corresponding critical values were t_α/2_ = 1.96 and t_β_ = 1.28. Patient satisfaction was selected as the primary outcome measure. Based on effect size estimates from relevant literature (10), the minimum required sample size was calculated to be 70 participants. Considering a 20% dropout rate, the final sample size was determined to be 88 cases.

Participants were allocated based on their admission order. A total of 44 breast cancer patients who underwent IBBR and were hospitalized in the Department of Breast Surgery at Zhejiang Provincial People’s Hospital from January to June 2024 were assigned to the control group. Another 44 patients meeting the same criteria, admitted between July and December 2024, were assigned to the intervention group. Relevant patient data were collected and entered using a double-check method by two independent researchers. Any discrepancies were promptly traced and corrected to ensure the accuracy and reliability of the dataset.

### 2.4 Integrated training model

#### 2.4.1 Development of the perioperative nursing protocol for IBBR in breast cancer patients

According to the “6S” evidence-based model (11), a top-down hierarchical literature search strategy was employed. The specific search terms, databases, as well as inclusion and exclusion criteria, are presented in **Supplementary Table 1**. Two members of the research team independently conducted a quality assessment of the selected literature. Consensus was reached based on the evaluation results, leading to the final inclusion of 16 articles (9, 12–26), comprising 2 clinical decision-making tools (15, 16), 7 clinical practice guidelines (9, 14, 17–19, 23, 25), 3 expert consensuses (12, 13, 20), 3 evidence summaries (21, 22, 24), and 1 systematic review (26). Based on the extracted evidence, the research team identified key elements related to clinical decision support, preoperative assessment, prophylactic antibiotic use, limb function exercises, health education, and postoperative evaluation. These elements were synthesized into a preliminary draft of the perioperative nursing protocol for patients undergoing IBBR, structured across three phases: preoperative, intraoperative, and postoperative care.

A panel of 20 nursing experts was invited to provide revision suggestions on the preliminary perioperative nursing protocol. Expert inclusion criteria, demographic characteristics, engagement levels, authority coefficient (Cr), and judgment coefficient (Ca) are presented in **Supplementary Table 2**. Following the first round of Delphi consultation, data were statistically analyzed. Indicators with mean scores ≥ 3.5 for both importance and feasibility, and a coefficient of variation (CV) ≤ 0.25, were retained. Expert feedback was reviewed item by item, and modifications to the questionnaire were made accordingly to generate the second-round survey. The Delphi process was concluded after consensus was reached in the second round. The Analytic Hierarchy Process (AHP) was employed to calculate the weight of each indicator level. Composite weights were determined using a stepwise multiplication method: composite weight = upper-level weight × current-level weight. These weights reflect the relative importance of each indicator within the overall system. A significance level of P < 0.05 was considered statistically significant. The final protocol consisted of three first-level indicators, 15 second-level indicators, and 46 third-level indicators. Detailed components of the finalized nursing protocol are presented in **Table 1**.

**Table 1:** Perioperative nursing protocol for IBBR in breast cancer patients.

Indicator	Importance		Feasibility		Weight	Composite weight
	Assignment (value, ± s)	CV	Assignment (value, ± s)	CV		
1. Preoperative Management	5.00	0	5.00	0	0.344	–
1.1 Patient Assessment	5.00	0	5.00	0	0.500	0.172
1.1.1 General Condition: This includes the patient’s age, obstetric history, height, weight, body mass index (BMI), smoking status, drug allergies, and comorbidities such as diabetes, connective tissue disorders, cardiovascular diseases, respiratory conditions, immune disorders, and mental health illnesses. A review of past anesthesia and surgical history is also conducted. If necessary, a multidisciplinary evaluation involving the departments of anesthesiology or nutrition may be arranged.	4.95 ± 0.22	0.05	4.75 ± 00.44	0.09	0.300	0.051
1.1.2 Tumor Characteristics: Size, location, local invasion, lymph node involvement, staging, pathology, and differentiation.	5.00	0	4.90 ± 0.44	0.09	0.300	0.051
1.1.3 Breast Morphology: Standardized photography in anterior and 45°/90° lateral positions using a digital camera under consistent lighting and background. Record volume, shape, symmetry, scars, and nipple-areola complex. A chaperone should be present.	4.9 ± 0.31	0.06	4.85 ± 0.37	0.08	0.290	0.050
1.1.4 Tissue Conditions: Skin integrity, scar adhesion, muscle and subcutaneous tissue quality, and vascular supply.	4.40 ± 0.89	0.20	4.50 ± 0.51	0.11	0.110	0.019
1.2 Decision Support	5.00	0	5.00	0	0.300	0.103
1.2.1 Decision-making capacity assessed via conflict scales. Provide patients with decision aids and information.	4.80 ± 0.41	0.09	4.50 ± 0.51	0.11	0.250	0.026
1.2.2 Shared Decision-Making: Includes risk-benefit analysis, reconstruction method, timing, and oncological safety.	4.70 ± 0.47	0.10	4.85 ± 0.49	0.10	0.240	0.025
1.2.3 Inform patients about aesthetic variability, the possibility of revision surgeries, and asymmetry in reconstructed breasts.	5.0	0	4.50 ± 0.51	0.11	0.260	0.027
1.2.4 Patients with large breasts (C cup or above), endocrine therapy, or radiation have increased complication risks.	4.90 ± 0.31	0.06	4.95 ± 0.22	0.05	0.250	0.026
1.3 Preoperative Preparation	4.80 ± 0.41	0.09	4.75 ± 0.44	0.09	0.200	0.069
1.3.1 Smoking cessation is advised 4 weeks before and 2 weeks after surgery.	4.85 ± 0.37	0.08	4.80 ± 0.62	0.13	0.250	0.017
1.3.2 It is recommended that obese patients with a BMI of ≥30 kg/m² implement a fat loss program.	4.40 ± 0.88	0.20	4.50 ± 0.51	0.11	0.200	0.014
1.3.3 Suspend tamoxifen/bevacizumab 3 weeks pre-op. Consult for anticoagulant use.	4.90 ± 0.31	0.06	4.80 ± 0.62	0.13	0.250	0.017
1.3.4 Use the “Reconstruction Expectations” module of the BREAST-Q pre-op to assess expectations.	4.65 ± 0.59	0.13	4.90 ± 0.45	0.09	0.150	0.010
1.3.5 Use anxiety and depression scales. Refer complex cases to mental health professionals.	4.80 ± 0.41	0.09	4.50 ± 0.51	0.11	0.150	0.010
1.3.6 Advise pre-op purchase of a suitable compression bra.	4.40 ± 0.94	0.21	4.50 ± 0.51	0.11	0.100	0.007
2. Intraoperative Management	4.60 ± 0.60	0.13	4.90 ± 0.31	0.06	0.316	–
2.1 Environment and Equipment	4.85 ± 0.37	0.08	4.75 ± 0.44	0.09	0.300	0.095
2.1.1 The circulating nurse should utilize laminar airflow or ultra-clean ventilation to reduce airborne transmission and surgical site contamination. Efforts should be made to minimize personnel movement through the operating room doors, with signage indicating “Breast Reconstruction with Implant in Progress” displayed outside.	4.85 ± 0.37	0.08	4.95 ± 0.22	0.05	0.500	0.048
2.1.2 Ensure that intraoperative equipment, including immediate specimen radiography and sentinel lymph node localization devices, is functioning properly and readily available.	4.70 ± 0.66	0.14	4.50 ± 0.51	0.11	0.500	0.048
2.2 Patient Positioning	4.80 ± 0.41	0.09	4.90 ± 0.31	0.06	0.300	0.095
2.2.1 Position the patient in the supine position with the affected upper limb abducted to 90° and securely fixed on an arm board. A soft pad should be placed under the shoulder and back to adequately expose the axillary region and prevent skin contact with the metal parts of the operating table.	4.90 ± 0.31	0.06	4.50 ± 0.51	0.11	0.400	0.038
2.2.2 Intraoperatively, elevate the upper body to at least a 60° semi-recumbent position to assess the shape, size, and symmetry of both breasts.	4.80 ± 0.52	0.11	5.0	0	0.300	0.029
2.2.3 When repositioning the patient, protect the surgical incision and surrounding skin, ensure all tubing remains patent and free from kinks or compression, and keep the linens dry and smooth to prevent pressure injuries.	4.70 ± 0.66	0.14	4.50 ± 0.51	0.11	0.300	0.029
2.3 Prophylactic Antibiotic Use	4.90 ± 0.31	0.06	4.95 ± 0.22	0.05	0.200	0.063
2.3.1 Administer systemic prophylactic antibiotics 1 hour prior to skin incision, as prescribed.	4.85 ± 0.37	0.08	5.0	0	0.400	0.025
2.3.2 Prior to the insertion of implants and/or meshes, soak them in an antimicrobial solution for 3–5 minutes.	4.90 ± 0.45	0.09	5.0	0	0.300	0.019
2.3.3 Assist the surgeon in irrigating the expander pocket with saline and an antibiotic solution after the expander is removed.	4.75 ± 0.64	0.13	4.95 ± 0.22	0.05	0.300	0.019
2.4 Hypothermia Prevention	4.90 ± 0.31	0.06	4.85 ± 0.37	0.08	0.200	0.063
2.4.1 Initiate pre-warming 30 minutes before surgery by maintaining the ambient room temperature at 24–26 °C, placing a warming blanket on the surgical bed, and preheating the equipment.	4.90 ± 0.31	0.06	4.50 ± 0.51	0.11	0.500	0.032
2.4.2 Continuously monitor the patient’s temperature intraoperatively through the postoperative phase. Maintain core body temperature at ≥36 °C using warming mattresses, circulating water garments, and fluid/blood warming devices.	4.80 ± 0.62	0.13	4.50 ± 0.51	0.11	0.500	0.032
2.5 Intraoperative Cooperation	4.90 ± 0.31	0.06	4.55 ± 0.51	0.11	0.200	0.063
2.5.1Confirm the specifications of implants and tissue expanders, as well as package integrity and valid period. Perform thorough visual examination to rule out scratches, defects and air bubbles. Operators shall try to keep these devices away from sharp instruments so as to lower the risk of device rupture.	5.0	0	5.0	0	0.400	0.025
2.5.2 Pass the implant or expander to the surgeon under strict aseptic conditions. Supervise the surgeon in changing gloves before handling the device, ensuring it does not come into contact with skin or breast flora.	4.80 ± 0.62	0.13	4.50 ± 0.51	0.11	0.300	0.019
2.5.3 Conduct a meticulous count of all instruments and dressings before and after cavity closure. Confirm accuracy before proceeding with layer-by-layer suturing.	4.80 ± 0.62	0.13	5.0	0	0.300	0.019
3. Postoperative Management	4.95 ± 0.22	0.05	4.95 ± 0.22	0.05	0.340	–
3.1 Flap Care	5.00	0	5.00	0	0.300	0.102
3.1.1 Immediately after surgery, apply a compression dressing to the superior and lateral aspects of the implant for fixation. Overly tight complete breast compression dressing is not recommended, given the potential risk of compromised blood supply to the nipple and cutaneous flap.	4.90 ± 0.31	0.06	5.0	0	0.400	0.041
3.1.2 The first 24 hours postoperatively are a critical observation period. Assess local flap perfusion and bleeding every 1–2 hours for signs such as cyanosis, fullness under or around the skin, crepitus, or local swelling and pain. Thereafter, perform assessments every 4–6 hours according to the patient’s condition, continuing until 72 hours postoperatively. Use Doppler ultrasound if necessary to evaluate blood flow, and promptly record and report any abnormalities.	4.90 ± 0.31	0.06	4.50 ± 0.51	0.11	0.300	0.031
3.1.3 At each nursing shift, inspect and record the color, character, and volume of the drainage fluid. Accurately document the total 24-hour drainage volume. Notify the physician immediately if bloody or turbid fluid is observed. Ensure unobstructed drainage, maintain the drainage bottle at a semi-negative pressure, and consider removing the drainage tube when the volume stabilizes and remains below 30 ml/day.	4.90 ± 0.31	0.06	4.95 ± 0.22	0.05	0.300	0.031
3.2 Bra Use	4.95 ± 0.22	0.05	4.95 ± 0.22	0.05	0.200	0.068
3.2.1 For patients with tissue expanders, it is recommended to begin wearing a shaping bra after the first saline injection. Continuous wear for 24 hours per day is required during the first week, followed by intermittent wear until one month after the final injection.	5.0	0	4.50 ± 0.51	0.11	0.400	0.027
3.2.2 For patients with implants, continuous wear of a compression bra and bandage is recommended for the first month postoperatively. From 1 to 3 months post-op, wearing only during the daytime is advised. After 3 months, switch to a well-fitted wireless bra. Patients are recommended to avoid underwire bras for approximately 6 months postoperatively, depending on individual wound healing conditions.	5.0	0	5.0	0	0.400	0.027
3.2.3Patients should be guided to evaluate the fitting degree of bras and local skin compression status. Excessive folds and persistent compression over the incision area should be avoided appropriately. It is advisable to adjust or replace the bra in a timely manner when obvious skin indentations or erythema occur.	4.90 ± 0.31	0.06	5.0	0	0.200	0.014
3.3 Affected Limb Mobility	4.90 ± 0.31	0.06	4.85 ± 0.37	0.08	0.200	0.068
3.3.1 Within 24 hours post-op, keep the affected shoulder adducted. ①Post-op days 1–3: perform fist clenching, finger extension, and wrist flexion exercises. ②Days 4–5: begin elbow flexion and extension exercises. ③Days 6–7: the affected arm should reach the same-side ear and opposite shoulder; use the healthy arm to assist in lifting movements. ④Days 8–14 (after drain removal and suture removal): initiate shoulder extension and other functional exercises.	4.70 ± 0.73	0.16	5.0	0	0.400	0.027
3.3.2 Rehabilitation training can be initiated 4–6 weeks after surgery under compression fixation. Patients are recommended to increase exercise intensity slowly, and refrain from stretches that induce pulling sensation or pain, as well as sudden arm raising movements.	4.90 ± 0.31	0.06	5.0	0	0.300	0.020
3.3.3 Most daily upper limb activities can be gradually resumed about 6 weeks postoperatively, and heavy lifting as well as overly intense exercises are suggested to be postponed appropriately.	4.80 ± 0.41	0.09	4.85 ± 0.37	0.08	0.300	0.020
3.4 Postoperative Evaluation	4.80 ± 0.41	0.09	4.80 ± 0.41	0.09	0.100	0.034
3.4.1 Assist the physician in taking standardized postoperative breast photos one day before discharge, under the same lighting and background as the preoperative photos, for comparison.	5.0	0	4.50 ± 0.51	0.11	0.500	0.017
3.4.2 At 1 month post-op, evaluate aesthetic outcome and patient satisfaction with the reconstructed breast using the Breast-Q questionnaire and Harries aesthetic scoring scale.	4.90 ± 0.31	0.06	4.50 ± 0.51	0.11	0.500	0.017
3.5 Implant Protection	4.90 ± 0.31	0.06	4.85 ± 0.37	0.08	0.100	0.034
3.5.1 Patients are advised to minimize severe impact and direct contact of sharp items against breast tissue, so as to lower the potential risk of implant damage.	4.90 ± 0.31	0.06	5.0	0	0.400	0.014
3.5.2 It is recommended that patients who undergo subpectoral implant placement minimize movements that activate or stretch the pectoralis major muscle, including chest expansion exercises and heavy weight lifting, within the initial 2–3 weeks postoperatively. In general, patients are suggested to limit intense upper limb exercises as well as excessive motions such as arm abduction, retroextension, chest expansion and shoulder shrugging in the first postoperative month, which can be adjusted according to individual wound recovery status.	4.90 ± 0.31	0.06	4.90 ± 0.31	0.06	0.300	0.010
3.5.3 It is recommended that patients minimize external compression on the reconstructed breast within the first three postoperative months. Supine sleeping position is highly preferred, while prolonged prone lying, prolonged lateral decubitus position and excessive head elevation should be avoided as far as possible. Patients are advised to consult clinicians promptly once obvious implant distortion, displacement or intolerable severe pain emerges, so as to reduce the incidence of subsequent adverse complications.	4.85 ± 0.37	0.08	5.0	0	0.300	0.010
3.6 Expander Care	5.0	0	4.90 ± 0.45	0.09	0.100	0.034
3.6.1 Inform patients with expanders that expansion typically begins 2 weeks post-op, with saline injections every 1–2 weeks at the clinic until the desired volume is achieved. During radiotherapy, saline should be temporarily removed to allow optimal exposure of the chest wall and internal mammary lymph nodes.	4.90 ± 0.31	0.06	5.0	0	0.400	0.014
3.6.2 After saline injection, temporary breast skin congestion and pain are normal. Keep the skin clean and avoid scratching the surface over the expander. If redness, swelling, heat, or pain occurs, seek medical attention promptly.	5.0	0	4.90 ± 0.45	0.09	0.300	0.010
3.6.3 Following tissue expansion, patients are recommended to minimize persistent local pressure, friction and physical impact. Sleeping on the healthy side is preferable, and excessively constrictive clothing is better avoided.	4.75 ± 0.44	0.09	5.0	0	0.300	0.010
3.7 Discharge Instructions	4.90 ± 0.31	0.06	4.90 ± 0.31	0.06	0.100	0.034
3.7.1 Rehabilitation: Provide patients with illustrated brochures explaining key points on implant protection and expander care, proper bra usage, functional exercise techniques, and dietary recommendations at home.	4.90 ± 0.31	0.06	4.55 ± 0.51	0.11	0.500	0.017
3.7.2 Follow-up: Conduct a telephone follow-up within one week after surgery. Recommend outpatient visits at 1 month, 3 months, 6 months, and 1 year post-op. Instruct patients to regularly assess implant status, rehabilitation progress, and psychological condition. Guide them in completing and filing the follow-up form.	4.90 ± 0.31	0.06	5.0	0	0.500	0.017

CV, Coefficient of variation.

#### 2.4.2 Design of the training program

The training program was systematically developed by the research team based on evidence-based literature and practical clinical needs. It incorporated three core components: model-based anatomical instruction, scenario-based simulation training, and hands-on clinical skills practice.

Model-based instruction utilized anatomical breast models and implant placement models to facilitate trainees’ visual and tactile understanding of the surgical process and relevant anatomical structures. Scenario-based training involved role-playing and situational problem-solving exercises designed around common clinical scenarios, including the recognition and management of flap cyanosis, early intervention for implant exposure, and patient education on postoperative functional rehabilitation. Practical training sessions were conducted in both real and simulated clinical environments, focusing on standardizing nurses’ performance in key perioperative tasks such as preoperative assessment, intraoperative collaboration, drainage management, and implant protection. These multimodal instructional strategies were designed to enhance the technical proficiency and communication competence of nursing staff across the perioperative continuum. The comprehensive training plan is detailed in **Table 2**.

**Table 2:** Training programs for healthcare workers’ care programs.

Day	Instructor(s)	Content	Training format	Participants
Day1-2	Director of Breast Surgery	Overview of breast cancer surgery and reconstruction; types of procedures and indications; implant-related complications	Didactic lecture	All nursing staff
Day3-4	Principal Investigator	Interpretation of the perioperative nursing protocol: preoperative assessment, intraoperative care, postoperative monitoring	PowerPoint based instruction	All nursing staff
Day5-6	Principal Investigator	Standardized procedures for implant protection and chest garment application, saline expansion protocol, and functional exercise guidance	Hands-on practice, model-based teaching	All nursing staff
Day7	Follow-up Coordinator & Principal Investigator	Standardized discharge education, follow-up planning, psychological support, and health education	Theoretical Tests; Scenario simulation Tests	All nursing staff

### 2.5 Intervention

The intervention group received refined management based on the integrated training model developed in this study, encompassing preoperative, intraoperative, and postoperative phases for patients undergoing IBBR. All nurses who delivered the intervention measures for patients in the intervention group received professional competency assessments, and all assessment results were qualified. The control group received the standard perioperative care protocol for breast reconstruction:(1) Preoperative care: Routine assessment, preoperative communication, preoperative skin preparation, and instructions regarding fasting; (2) Intraoperative care: Standard positioning, with empirical cooperation between scrub and circulating nurses; (3) Postoperative care: Routine surgical nursing, flap monitoring, and health education.

### 2.6 Assessment and outcomes

#### 2.6.1 Chinese version of the BREAST-Q 2.0 reconstruction module questionnaire

This questionnaire (27) consists of six dimensions; however, based on the needs of this study, only three dimensions were selected: satisfaction with breasts (0–100 points), satisfaction with surgical outcomes (0–100 points), and satisfaction with medical care (0–100 points). Higher scores indicate greater patient satisfaction.

#### 2.6.2 Postoperative complications

The Clavien-Dindo classification was used to grade postoperative complications (28).Complications occurring within 30 days after surgery, including bleeding, seroma, infection, flap necrosis and implant removal, were identified by reviewing the medical records and postoperative pictures maintained by the surgeons. We dichotomized postoperative complications into grades ≤ II and ≥ III for statistical analysis.

### 2.7 Data collection

Participants completed the BREAST-Q questionnaire either electronically or in paper form based on their preference at 30 days postoperatively, and reported any additional unplanned surgical interventions, especially unplanned implant removal.

### 2.8 Statistical analysis

Statistical analysis was performed using SPSS 22.0 software. Continuous variables were expressed as mean ± standard deviation, while categorical data were presented as frequencies, percentages, or proportions. Comparisons were conducted using the chi-square test, the rank-sum test or Mann-Whitney U test. Expert ratings on the importance and feasibility of each item were presented as mean ± standard deviation ( $\bar{x}$ ± s), and the coefficient of variation was used to represent the degree of consensus among experts for each item. The analytic hierarchy process (AHP) was used to calculate the weights of each level of indicators. The combined weight was calculated using the layer-by-layer multiplication method: combined weight = weight of the higher-level indicator × weight of the current-level indicator. This reflects the relative importance of each indicator within the overall system.

## 3 Results

### 3.1 Comparison of general characteristics

**Figure 1:**
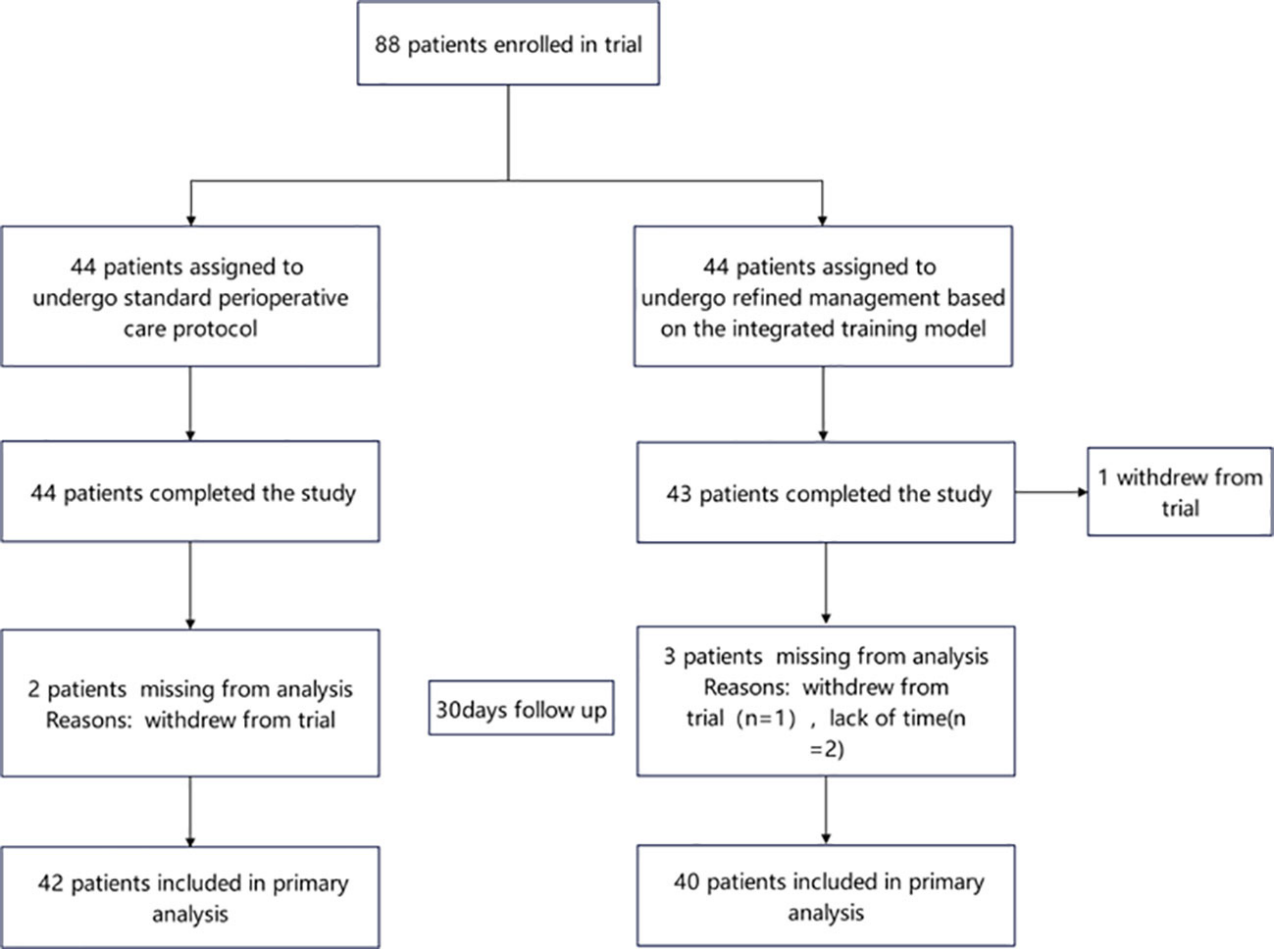
The CONSORT flow diagram.

A total of 40 cases in the intervention group and 42 cases in the control group completed the study (**Figure 1**). There were no statistically significant differences in the general characteristics between the two groups (*P* > 0.05), as shown in **Table 3**.

**Table 3:** Comparison of general characteristics between the two groups.

Variable	Control group (n = 42)	Experimental group (n = 40)	p-value
Age (years), mean (SD)	46.15(6.78)	45.21(8.89)	0.593
BMI(kg/m^2^)			0.518
<18.5	3((7.1%)	1(2.5%)	
18.5~24.9	33(78.6%)	35(87.5%)	
≥25.0	6(14.3%)	4(10.0%)	
Educational level			0.725
Primary school or below	5(11.9%)	4(10.0%)	0.725
Junior high school	14(33.3%)	10(25.0%)	
Senior high school	13(30.9%)	17(42.5%)	
College or above	10(23.8%)	9(22.5%)	
Marital status			0.586
Married	40(95.2%)	39(97.5%)	
Divorced or widowed	2(4.8%)	1(2.5%)	
Smoking history			0.747
Never	38(90.5%)	35(87.5%)	
Past	4(9.5%)	4(10.0%)	
Current	0(0.0%)	1(2.5%)	
Preoperative neoadjuvant chemotherapy			0.604
Yes	2(4.8%)	3(7.5%)	
No	40(95.2%)	37(92.5%)	
History of chest radiation			0.659
Yes	24(57.1%)	19(47.5%)	
No	18(42.9%)	21(52.5%)	
Diabetes			0.286
Yes	9(21.4%)	5(12.5%)	
No	33(78.6%)	35(87.5%)	
Type of breast reconstruction			0.865
Immediate	15(35.7%)	15(37.5%)	
Delayed	27(64.3%)	25(62.5%)	
Type of mastectomy			0.191
Nipple Sparing Mastectomy	28(66.7%)	21(52.5%)	
Skin Sparing Mastectomy	14(33.3%)	19(47.5%)	
Axillary surgery			0.124
Sentinel lymph node biopsy	29(69.0%)	21(52.5%)	
Axillary lymph node dissection	13(31.0%)	19(47.5%)	
Use of mesh			0.126
Yes	28(66.7%)	20(50.0%)	
No	14(33.3%)	20(50.0%)	
Type of breast implant			0.865
Tissue expander	27(64.3%)	25(62.5%)	0.865
Permanent breast implant	15(35.7%)	15(37.5%)	
TNM stage			0.983
Stage II	54.8%(23)	55.0%(22)	
Stage III	45.2%(19)	45.0%(18)	
Type of breast implant			0.852
Implant plane			0.852
Subpectoral	59.5%(25)	57.5%(23)	
Prepectoral	40.5%(17)	42.5%(17)	
Implant volume(ml), mean (SD)	235.82(87.26)	247.65(90.23)	0.617
Affected side			0.275
Left	25(59.5%)	19(47.5%)	
Right	17(40.5%)	21(52.5%)	

BMI, body mass index; TNM, Tumor-Node-Metastasis Staging System.

### 3.2 Comparison of satisfaction between the two groups

The experimental group reported significantly higher scores in satisfaction with breast appearance, satisfaction with surgical outcomes, and satisfaction with medical care compared to the control group (*P* < 0.05), as shown in **Table 4**.

**Table 4:** Comparison of satisfaction between the two groups.

Outcomes	Control group (n = 42)	95%CI	Experimental group (n = 40)	95%CI	*p*-value
Satisfaction with Breast Appearance,mean (SD)	80.32(4.97)	78.82-81.82	85.37(5.45)	83.68-87.06	<0.001
Satisfaction with Surgical Outcomes,mean (SD)	79.23(5.21)	77.65-80.81	83.66(5.70)	81.89-85.43	<0.001
Satisfaction with Medical Care,mean (SD)	83.56(5.99)	81.75-85.37	88.72(6.28)	86.77-90.67	<0.001

### 3.3 Comparison of complication rates between the two groups

The 30-day overall postoperative complication rate was 9.8% (8/82). The complication incidence was 14.3% (6/42) in the control group and 5.0% (2/40) in the experimental group. Although a higher complication proportion was observed in the control group, no statistically significant difference was detected between the two groups (P>0.05), as presented in **Table 5**.The distribution of Clavien-Dindo complication grades showed no statistically significant difference between the control group and the experimental group (Mann-Whitney U test, U = 920.0, Z = 1.44, P = 0.152), as presented in **Table 6**. All complications were managed following the standardized clinical protocol of our center, with uniform treatment principles for both groups. Two patients in the control group underwent reoperation owing to flap necrosis and hemorrhagic hematoma respectively, and both recovered well. No reoperation occurred in the experimental group.

**Table 5:** Postoperative complications in the two groups.

Complications	Control group (n = 42)	Experimental group (n = 40)	*p*-value
Any complication			0.265
Yes	6	2	
NO	36	38	
Infection			0.241
Yes	3	0	
NO	39	40	
Bleeding			1.000
Yes	1	1	
NO	41	39	
Seroma			1.000
Yes	1	1	
NO	41	39	
Flap necrosis			1.000
Yes	1	0	
NO	41	40	
Implant removal			1.000
Yes	0	0	
NO	42	40	
Reoperation			
Yes	2	0	0.494
NO	40	40	

**Table 6:** Proportions of complication grades in the two groups.

Clavien Dindo classification	Control group (n = 42)		Experimental group (n = 40)		p-value
	N	%	N	%	
None	36	85.7%	38	95%	0.152
Grade ≤ II	4	9.5%	2	5.0%	
Grade ≥ III	2	4.8%	0	0%	

## 4 Discussion

### 4.1 Integrated training enhances patient satisfaction after IBBR

This quasi-experimental study found that patients in the intervention group reported significantly higher scores in breast satisfaction, surgical outcome satisfaction, and care satisfaction compared to the control group (*P* < 0.001). These results suggest that the integrated training plan—delivered through a structured perioperative nursing protocol—effectively enhanced the overall care experience for patients undergoing IBBR.

Several factors may have contributed to the observed improvement in patient satisfaction. First, the training intervention equipped nursing staff with standardized procedures and clear operational guidelines, reducing inconsistencies in care delivery (29). Second, enhanced communication between nurses and patients, supported by structured education sessions, likely improved patient understanding and expectations about the reconstruction process, which is known to influence perceived satisfaction. Third, nurses who participated in hands-on simulations and scenario-based training may have developed stronger technical confidence and empathy, translating into more attentive and responsive care behaviors at the bedside.

The training program implemented in this study integrated multiple instructional strategies aimed at enhancing nurses’ clinical competence and the consistency of care delivery (7). During the training process, anatomical breast and implant models were utilized to visually demonstrate structural relationships and surgical pathways, enabling nurses to develop a clearer understanding of anatomy and operative procedures. Standardized clinical scenarios (30)—such as recognition of postoperative flap cyanosis, early management of implant exposure, and patient education on functional rehabilitation—were incorporated through role-play and scenario-based simulations to improve nurses’ clinical responsiveness and communication skills (31).

In addition, nurses were divided into small groups to engage in hands-on, standardized training within real or simulated environments, covering key components such as preoperative assessment, intraoperative cooperation, postoperative drainage management, and implant protection. The findings of this study indicate that integrating comprehensive training with a standardized nursing protocol can significantly improve the precision and consistency of nursing services. In complex surgical contexts such as IBBR, coordinated care and empathetic communication are critical to promoting patient recovery and satisfaction (32).

### 4.2 Integrated training enhances patient satisfaction after IBBR

The results of this study showed that the complication rate in the intervention group was 5.0%, compared to 14.3% in the control group. Although this reflects a downward trend, the difference was not statistically significant (*P* = 0.148). This lack of significance may be attributed to the relatively small sample size, which was influenced by the study’s inclusion and exclusion criteria. As a result, the statistical power was insufficient to detect between-group differences, even if the intervention potentially had a positive clinical effect. Nevertheless, the findings suggest that the integrated training program may hold clinical value in reducing postoperative complications. Future studies with larger sample sizes or multi-center designs are recommended to further evaluate the effectiveness of combining the training plan with perioperative nursing protocols.

### 4.3 The constructed nursing protocol and training pathway demonstrate clinical applicability

The nursing protocol developed in this study was constructed based on an extensive evidence base from the literature, combined with the Delphi method and Analytic Hierarchy Process (AHP), ensuring both scientific validity and practical relevance (33). The protocol covers the full perioperative care continuum—from preoperative assessment and psychological preparation to postoperative monitoring and discharge guidance. Each component was operationalized into specific, measurable actions, addressing previous issues of reliance on subjective nursing experience and vague observational standards, thereby enhancing the clarity and reproducibility of clinical practice.

The integrated training program was designed around key management nodes throughout the preoperative, intraoperative, and postoperative phases. Centralized training sessions improved nurses’ ability to implement the protocol, reflecting a clear “evidence–translation–practice” logic pathway. This multimodal approach contributed to the standardization of care delivery among surgical nurses and reduced variability in critical tasks such as flap monitoring, drainage volume management, and patient education. Moreover, involving patients in some educational activities fostered mutual understanding and promoted greater patient engagement. By aligning the knowledge and expectations of both care providers and recipients, the intervention helped create a more cohesive and responsive care environment.

Overall, this structured protocol and comprehensive training model address known challenges in IBBR nursing, including inconsistent practices, communication barriers, and lack of coordination across care phases. Future studies should consider expanding the training scope and extending the follow-up period, while incorporating long-term outcomes such as quality of life and psychological adaptation to comprehensively assess the sustainability and impact of the intervention.

## 5 Limitations

Several limitations should be acknowledged. First, the quasi-experimental design and single-center setting may limit the generalizability of the findings. The lack of randomization introduces a potential risk of selection bias, which could undermine the internal validity of the results. Furthermore, the relatively small sample size may have reduced the statistical power, particularly with respect to detecting differences in postoperative complications. Additionally, the follow-up period was relatively short, thereby precluding the assessment of long-term outcomes such as implant failure, capsular contracture, and patient-reported quality of life. Moreover, the cost-effectiveness of the integrated training program and nursing protocol was not evaluated, which could be crucial for guiding broader clinical implementation. Future multicenter randomized controlled trials with larger sample sizes and extended follow-up periods are necessary to corroborate and expand upon these findings.

## 6 Conclusion

This study revealed that standardized perioperative nursing combined with targeted health education significantly improves overall patient satisfaction following IBBR. The intervention group reported better satisfaction regarding breast cosmetic outcomes, surgical results, and nursing experience than the control group. Although a lower postoperative complication rate was observed in the intervention group, the difference was not statistically significant, possibly due to the limited sample size. As a single-center study with non-randomized time-phase grouping and a short follow-up duration, the present findings only provide preliminary clinical evidence. The established nursing and health education protocols help standardize perioperative nursing workflows and improve patients’ medical experience. Large-sample, multicenter, long-term randomized controlled trials are required to further validate the long-term efficacy and complication prevention effects of this nursing strategy. Overall, this standardized model offers a credible reference for clinical nursing practice in IBBR.

## Data Availability

The raw data supporting the conclusions of this article will be made available by the authors, without undue reservation.

## Supplementary material

Supplementary Table 1 Literature search strategy.

Supplementary Table 2 Delphi expert consultation.
