# Supplementary material for: The effectiveness of combined training model in perioperative nursing for implant-based breast reconstruction: a quasi-experimental study
Source: Front Oncol. 2026 Jun 15;16:1794390. doi: 10.3389/fonc.2026.1794390 (PMC13310678; doi:10.3389/fonc.2026.1794390)
Supplement: Supplementary Table 1 — Literature search strategy. [file DataSheet1.pdf]

1.Search terms

1.1 Chinese search term: “乳房切除术/乳腺癌/乳腺恶性肿瘤 ““乳房重建/乳房再造/扩张器/假体/植入物” “康复/护理/照护/管理”“指南/专家共识/最佳实践/证据总结/meta 分析/荟萃分析/系统综述/系统评价”

1.2 English search terms: “breast neoplasm /breast tumor/breast cancer /mammary cancer/ breast malignant neoplasm/breast carcinoma /breast malignant tumor ” “ tissue expander/ breast implant/tissue expansion device/internal breast prosthes/ breast implantation/ mammaplasty /breast reconstruction” “guideline/guide/guidance/consensus/systematic review/meta-analysis”

2.Databases

Joanna Briggs Institute, UpToDate, PubMed, Web of Science, Embase, Cochrane Library, CHINAL, NICE, NCCN, GIN, Medive.

3.Inclusion and exclusion criteria

3.1 Inclusion criteria:

(1) Literature types included guidelines, expert consensus statements, recommended practices, evidence summaries, and systematic reviews;

(2) The study population consisted of breast cancer patients undergoing implant-based breast reconstruction;

(3) The content covered decision-making support for implant-based breast reconstruction, preoperative assessment, prophylactic antibiotic use, health education, functional exercise of the affected limb, and postoperative evaluation;

(4) Publications were in Chinese or English.

3.2 Exclusion criteria:

(1) Interpretations or translated versions of international guidelines;

(2) Outdated versions of guidelines that have already been updated;

(3) Literature for which the full text was not available.
