# Supplementary material for: The effectiveness of combined training model in perioperative nursing for implant-based breast reconstruction: a quasi-experimental study
Source: Front Oncol. 2026 Jun 15;16:1794390. doi: 10.3389/fonc.2026.1794390 (PMC13310678; doi:10.3389/fonc.2026.1794390)
Supplement: Supplementary Table 2 — Delphi expert consultation. [file DataSheet2.pdf]

1. Expert inclusion criteria

The purposive sampling method was used to select experts based on the following inclusion criteria:

(1) Professionals with more than 10 years of experience in perioperative management, clinical teaching, or research related to implant-based breast reconstruction for breast cancer patients in tertiary grade A hospitals;

(2) Holding a bachelor's degree or above;

(3) Medical experts with associate senior titles or above, and nursing experts with head nurse titles or above, possessing extensive clinical experience and strong research capabilities, able to provide comprehensive and valuable insights for this study;

(4) Willingness to voluntarily participate in the study.

2. General Information on Experts

A total of 20 experts from provincial or municipal hospitals in Zhejiang, Hubei, Shanghai, and Tianjin were ultimately selected to participate in two rounds of Delphi consultation. The experts had a mean age of 46.72 ± 5.47 years and an average of 15.33 ± 4.66 years of professional experience. Among them, 3 held doctoral degrees (15.0%), 7 held master's degrees (35.0%), and 10 held bachelor's degrees (50.0%). There were 11 experts with senior professional titles (55.0%) and 9 with intermediate titles (45.0%). In terms of professional roles, 4 (20.0%) were clinical medical experts, 15 (75.0%) were clinical nursing experts, and 1 (5.0%) was a nursing education expert.

3. Expert engagement and authority level

In both rounds of the study, 20 consultation questionnaires were distributed and all were validly returned, resulting in a 100% effective response rate and a 100% completion rate. This indicates a high level of engagement and commitment from the participating experts. The expert authority coefficients in the two rounds were as follows: Cs = 0.818 and 0.865, Ca = 0.888 and 0.912, and Cr = 0.853 and 0.888. Since the Cr values in both rounds were ⩾ 0.700, the experts were considered highly authoritative, and the reliability of the Delphi consultation results was deemed

strong.

4. Consensus Level Among Expert Opinions

The expert opinions from the two rounds of Delphi consultation demonstrated a high degree of consistency, as shown in Table. The coefficient of variation for all levels of indicators ranged from 0 to 0.212.

Table. Kendall’s Coefficient of Concordance for Two Rounds of Expert Consultation

Round	Importance of Primary Indicators			Importance of Secondary Indicators			Importance of Tertiary Indicators		
	W	χ^2	*P*	W	χ^2	*P*	W	χ^2	*P*
Round 1	0.221	8.857	< 0.05	0.276	77.195	< 0.01	0.203	203.304	< 0.01
Round 2	0.269	10.750	< 0.01	0.352	98.491	< 0.01	0.290	290.003	< 0.01
